# Low-Density Lipoprotein Cholesterol Treatment Target Achievement in Patients with Myocardial Infarction, Percutaneous Coronary Intervention, or Stroke in Hong Kong

**DOI:** 10.31083/j.rcm2310327

**Published:** 2022-09-26

**Authors:** Joseph E Blais, Vincent KC Yan, Jiaxi Zhao, Celine SL Chui, Ian CK Wong, Chung Wah Siu, Esther W Chan

**Affiliations:** ^1^Centre for Safe Medication Practice and Research, Department of Pharmacology and Pharmacy, LKS Faculty of Medicine, University of Hong Kong, Hong Kong SAR, China; ^2^School of Public Health, LKS Faculty of Medicine, University of Hong Kong, Hong Kong SAR, China; ^3^Digital and Data Innovation, AstraZeneca Global R&D (China) Co, Ltd, 200000 Shanghai, China; ^4^School of Nursing, LKS Faculty of Medicine, University of Hong Kong, Hong Kong SAR, China; ^5^Laboratory of Data Discovery for Health, Hong Kong Science Park, Sha Tin, Hong Kong SAR, China; ^6^Aston Pharmacy School, Aston University, B4 7ET Birmingham, UK; ^7^Division of Cardiology, Department of Medicine, University of Hong Kong, Hong Kong SAR, China; ^8^Department of Pharmacy, The University of Hong Kong-Shenzhen Hospital, 518000 Shenzhen, GuangDong, China; ^9^The University of Hong Kong Shenzhen Institute of Research and Innovation, 518000 Shenzhen, GuangDong, China

**Keywords:** low-density lipoprotein cholesterol, myocardial infarction, stroke, transient ischaemic attack, percutaneous coronary intervention, secondary prevention, statins, Hong Kong

## Abstract

**Background:**

Elevated concentrations of low-density lipoprotein cholesterol (LDL-C) are an important cause of recurrent cardiovascular events. This study aimed to describe the distribution and achieved concentrations of LDL-C among patients with myocardial infarction (MI), percutaneous coronary intervention (PCI), stroke, or transient ischaemic attack (TIA) in Hong Kong.

**Methods:**

Patients with a lipid test from a public hospital were identified from the Clinical Database and Analysis Reporting System of the Hong Kong Hospital Authority. Among patients with an inpatient hospitalization for MI, PCI, stroke or TIA, between 2003 to 2016, the distribution of LDL-C levels and the number (%) of patients achieving an absolute concentration of LDL-C <1.8 mmol/L at baseline (in-hospital) and during 12 months after hospital discharge were described.

**Results:**

A total of 18417 patients were included (mean [SD] age, 70.0 [12.9] years; male, 60.3%), of which 3637 had MI, 4096 had PCI, and 10684 had stroke or TIA. At hospital discharge 12082 (65.6%) patients were prescribed statins, 690 (3.7%) were prescribed nonstatins, and 1849 (10.0%) achieved an LDL-C <1.8 mmol/L. Overall, 5654 (30.7%) patients did not have LDL-C result available within 12 months of discharge (MI, 605 [16.6%]; PCI, 432 [10.5%]; stroke or TIA, 4617 [43.2%]). Among the overall cohort, 4591 (24.9%) patients achieved an LDL-C <1.8 mmol/L during 12 months of follow-up (MI, 1288 [35.4%]; PCI, 1542 [37.6%]; stroke or TIA, 1761 [16.5%]). Improvements in achieved LDL-C were observed over time with a mean LDL-C 2.64 (0.92) mmol/L and 20.0% of patients achieving an LDL-C <1.8 mmol/L in 2003 as compared with a mean LDL-C 1.86 (0.70) mmol/L and 53.9% of patients achieving an LDL-C <1.8 mmol/L in 2016.

**Conclusions:**

In this single centre cohort study from Hong Kong, nearly half of patients with MI, PCI, or stroke in 2016 appear to qualify for intensification of lipid-modifying drug treatment in order to achieve a treatment goal of LDL-C <1.8 mmol/L. Further research is required in Hong Kong to assess contemporary management of LDL-C in a larger group of patients with established atherosclerotic cardiovascular disease.

## 1. Introduction

Despite the availability and affordability of statins, a large proportion of high risk individuals in Asia have low-density lipoprotein cholesterol (LDL-C) levels that remain above recommended treatment targets which contributes to the burden of atherosclerotic cardiovascular disease (ASCVD) [1, 2, 3]. Current guidelines from the American College of Cardiology/American Health Association and European Society of Cardiology/European Atherosclerosis Society (ESC/EAS) emphasize achieving an absolute LDL-C <1.8 mmol/L for people with ASCVD or who are at high risk of ASCVD events [4, 5].

Further reductions in LDL-C are now obtainable with the addition of ezetimibe or proprotein convertase subtilisin/kexin type 9 (PCSK9) inhibitors to statin therapy. These nonstatin lipid-modifying drugs improve cardiovascular outcomes in secondary prevention of cardiovascular events [6, 7, 8], and are recommended for patients who do not achieve LDL-C targets on maximally tolerated doses of statins [4]. Guideline recommended nonstatin therapies have long been available in Hong Kong: ezetimibe (approved July 2003), evolocumab (approved May 2016), and alirocumab (approved October 2016) [9]. However, underuse of both statin and nonstatin lipid-modifying drugs in people with acute coronary syndrome (myocardial infarction (MI) or unstable angina) is a challenge in Hong Kong public hospitals: research conducted between 2009 to 2015 indicates that 25% of individuals with acute coronary syndrome did not receive statins by discharge and there was limited use of ezetimibe [10].

Emerging evidence suggests that achieving LDL-C treatment targets is also associated with improve cardiovascular outcomes in individuals with recent percutaneous coronary intervention (PCI) [11] and stroke or transient ischaemic attack (TIA) [12, 13]. In the Stroke Prevention by Aggressive Reduction in Cholesterol Levels (SPARCL) trial, atorvastatin 80 mg daily compared with placebo reduced the risk of fatal or nonfatal stroke and overall vascular events in individuals with a recent stroke or TIA and an LDL-C of 2.6–4.9 mmol/L [12, 14]. Targeting an LDL-C <1.8 mmol/L versus a higher target of 2.3–2.8 mmol/L with a statin, ezetimibe, or both, in individuals with ischaemic stroke or TIA in the Treat Stroke to Target (TST) trial also reduced the risk of cardiovascular events [13]. A comprehensive description of achieved LDL-C levels, that includes the early period of PCSK9 inhibitor availability, is needed in Hong Kong. Therefore, we aimed to describe the distribution of LDL-C concentrations at baseline and during one year of follow-up in patients hospitalized for a MI, PCI, stroke, or TIA.

## 2. Methods

### 2.1 Study Design and Setting

We did a cohort study using electronic health record data from the Hong Kong Hospital Authority. The Hospital Authority is the statutory body responsible for public healthcare in Hong Kong; its hospitals have about 80% of the region’s hospital beds [15]. We extracted data from the Clinical Data and Administrative Reporting System (CDARS), to initially identify a cohort of patients who had a lipid test from 1 January 2004 to 3 March 2014 at the Queen Mary Hospital—the major acute care and specialist outpatient hospital within the Hong Kong West Cluster. The catchment area of the Queen Mary Hospital is the central and western part of Hong Kong Island. This geographic region includes approximately 7% of the Hong Kong population. CDARS contains records of diagnoses, medication dispensing, hospital admission and discharge, procedures, demographics, and laboratory tests. This study was approved by the Hong Kong West Cluster/HKU Institutional Review Board (Reference Number UW 14-334).

### 2.2 Eligibility Criteria

We defined the index date (time zero) as the earliest discharge date of an inpatient diagnosis or procedure diagnosis ranking in the first through third position, for MI, stroke or TIA, or PCI, between 1 January 2003 to 31 December 2016. Next, we excluded patients with a date of death on or before the index date, those who did not have at least one LDL-C test result during the index hospitalization (admission date to discharge date inclusive) or during 365 days after hospital discharge, and those aged <18 years on the index date.

### 2.3 Baseline Period and Variables

We used several time windows to assess baseline variables (**Supplementary Table 1**). We used a one year look-back window to assess most baseline diagnoses, medication use, and laboratory tests. Exceptions included a prior history of MI, stroke or TIA, PCI, and coronary artery bypass graft surgery (CABG), for which we looked back until the start of all diagnosis and procedures data availability. We included descriptive variables, those required to calculate the TIMI (Thrombolysis in Myocardial Infarction) Risk Score for Secondary Prevention (TRS 2ºP), and those judged to be important confounders (**Supplementary Table 1**).

### 2.4 Laboratory Tests

We inspected the distribution of each laboratory test and removed results with missing numeric values. Because LDL-C is calculated according to the Friedewald formula, we excluded any test results with values less than zero or reported as unfit for calculating LDL-C due to triglycerides >4.5 mmol/L. All other lab tests appeared to have biologically plausible values. If non–HDL-C was not reported, it was calculated as T⁢o⁢t⁢a⁢l⁢c⁢h⁢o⁢l⁢e⁢s⁢t⁢e⁢r⁢o⁢l-H⁢D⁢L⁢c⁢h⁢o⁢l⁢e⁢s⁢t⁢e⁢r⁢o⁢l. We were interested in lipid levels during the index hospitalization thus the baseline exposure assessment window for lipid tests was from the date of hospital admission to the date of discharge.

### 2.5 Index Event

For patients with multiple index diagnoses or procedures, we classified the index event for each patient into one of three mutually exclusive groups in hierarchical order: first as MI, second as stroke or TIA, and third as PCI.

### 2.6 Medications

Medication classes were identified using British National Formulary sections and specific medications were identified using drug item codes (**Supplementary Table 1**). Statins were classified into low-, moderate-, and high-intensity according to their average anticipated reduction in LDL-C [16]. We defined nonstatin lipid-modifying drugs as ezetimibe, fibrates, bile acid sequestrants, and PCSK9 inhibitors.

### 2.7 TIMI Risk Score for Secondary Prevention

The TRS 2ºP uses nine clinical risk factors to estimate the risk of cardiovascular death, myocardial infarction, and ischaemic stroke in patients with a history of acute coronary syndrome [17]. Risk categories have been defined as low (0 to 1 risk factors), intermediate (2 risk factors), and high (≥3 risk factors). For the risk factor of estimated glomerular filtration rate (eGFR) <60 mL/min, we classified individuals first based on their measured creatinine clearance, if available (creatinine clearance <60 mL/min). If creatinine clearance was not available, we calculated eGFR using serum creatinine and the Modification of Diet in Renal Disease (MDRD) Study equation [17]. Finally, if we could not calculate eGFR, patients with a prior diagnosis of renal disease were classified as having the eGFR <60 mL/min risk factor.

We defined the hypertension risk factor as having a diagnosis of hypertension based on diagnosis codes or a baseline prescription for any antihypertensive medication. Similarly, we defined the diabetes mellitus risk factor as having a diagnosis of diabetes mellitus or a prescription for an antidiabetic medication. Each TRS 2ºP risk factor contributed a weight of one, and the total number of risk factors at baseline were summed. The remaining risk factors that are not derived from other variables are the same as those in **Supplementary Table 1** (e.g., smoker, peripheral artery disease). We described TRS 2ºP risk factors on a continuous scale and categorically (0, 1, 2, and ≥3 risk factors).

### 2.8 Exposure Assessment

Baseline (in-hospital) LDL-C was the primary exposure of interest and its distribution was presented continuously and categorically. We classified LDL-C in two ways. First, we created five categories: <1.8, 1.8–2.6, 2.7–3.9, ≥4 mmol/L, and missing. Second, we categorized LDL-C into three categories: <1.8, ≥1.8 mmol/L, or missing. A target LDL-C of 1.8 mmol/L was selected in accordance with guideline recommendations for very high-risk secondary prevention patients at the time of starting our study [4, 18].

### 2.9 Follow-Up Period and Outcome Assessment

The follow-up window for all outcomes was defined as one year after hospital discharge. Follow-up LDL-C levels were assessed in five time windows: 30, 90, 180, and 365 days; and any time during the one year follow-up window. If a patient had multiple LDL-C results during the window, we selected the latest results farthest away from index date. For each follow-up window, we described the distribution of LDL-C for the overall cohort and stratified by index event. For patients with at least one baseline and one follow-up LDL-C level, we calculated the percent reduction in LDL-C using the latest LDL-C level one year after hospital discharge, defined as $\frac{F\,o\,l\,l\,o\,w-u\,p\,L\,D\,L-C-b\,a\,s\,e\,l\,i\,n\,e\,L\,D\,L-C}{b\,a\,s\,e\,l\,i\,n\,e\,L\,D\,L-C}\times 100\%$.

### 2.10 Statistical Analysis

Patient characteristics and LDL-C are presented as numbers and percentages, means and standard deviations (SD), or medians and interquartile ranges (IQR), as appropriate. We visualized the distribution of LDL-C within each follow-up window according to index diagnosis using box plots. Achievement any time after discharge of LDL-C <1.8 mmol/L, reduction ≥50% LDL-C from baseline, LDL-C <1.8 mmol/L and ≥50% reduction from baseline, mean (SD) achieved absolute LDL-C, and mean relative (%) reductions in LDL-C from baseline were assessed by calendar year. Two authors (JEB and VKCY) independently conducted the analysis using R software version 3.6.1 (R Core Team; Vienna, Austria).

## 3. Results

After application of the exclusion criteria, we included a total of 18417 patients with a diagnosis of MI, stroke or TIA, or PCI, between 1 January 2003 to 31 December 2016 (Fig . 1). Baseline characteristics of the included patients are shown Table 1. The majority of patients were male, Chinese, diagnosed with an index stroke or TIA while 56.4% had a diagnosis of hypertension and 31.9% had a diagnosis of diabetes. By hospital discharge 66.8% were prescribed a lipid-modifying drug. Statins were the most prescribed class of lipid-modifying drug and 70.0% of patients prescribed statins received a moderate-intensity statin.

**Fig. 1.**
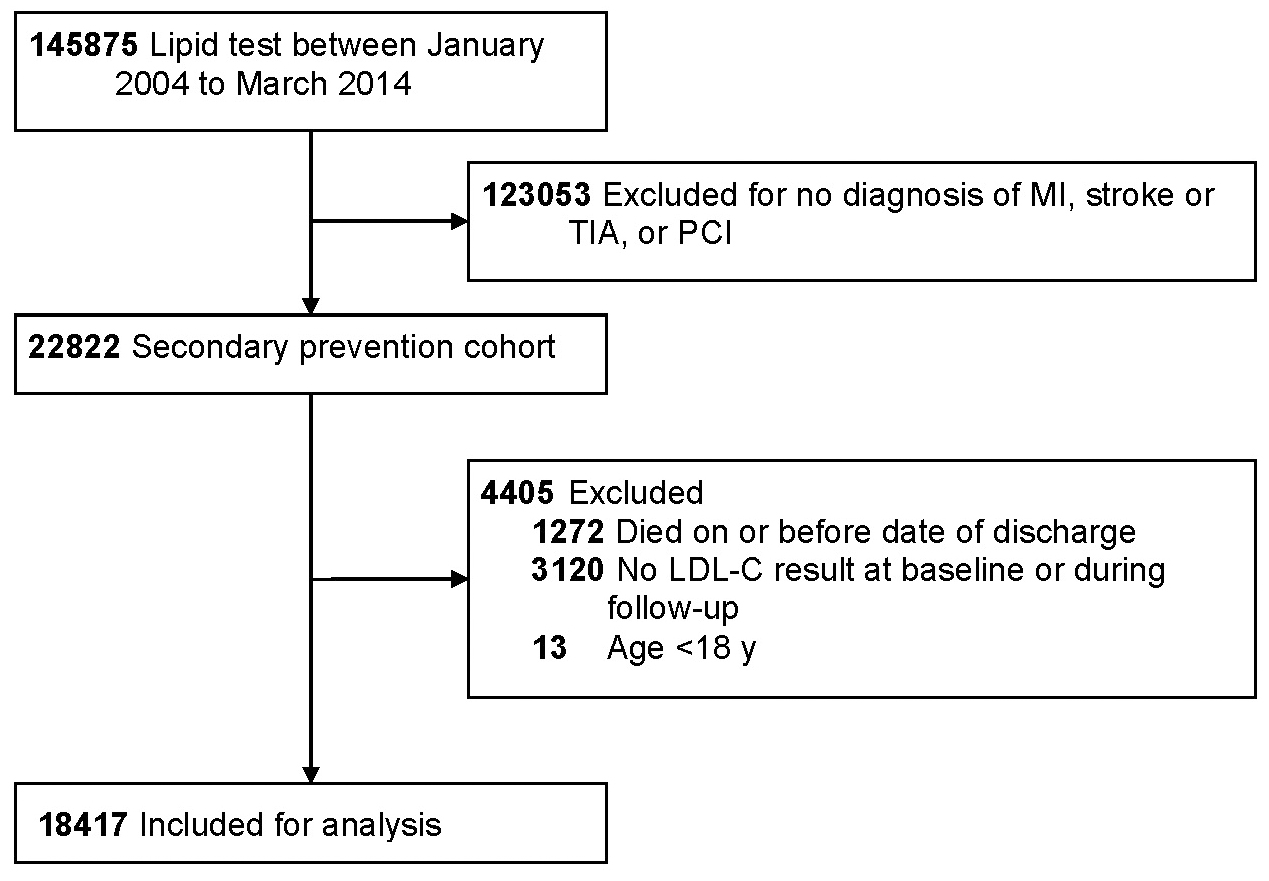
**Flowchart of patients who had a lipid test at the Queen Mary Hospital**. MI, myocardial infarction; PCI, percutaneous coronary intervention; TIA, transient ischaemic attack.

**Table 1.** **Baseline characteristics of the included patients discharged with a diagnosis of a myocardial infarction, stroke, transient ischaemic attack, or percutaneous coronary intervention**.

Characteristic		Patients (n = 18417)
Demographics		
Male (%)		11102 (60.3)
Age, years (mean (SD))		70.0 (12.9)
Age ≥75 years (%)		7429 (40.3)
Nationality (%)		
	Chinese	17170 (93.2)
	Other	462 (2.5)
	Missing	785 (4.3)
Hospital Authority cluster of residence (%)		
	HKW	12912 (70.1)
	HKE	2518 (13.7)
	KW	1174 (6.4)
	KE	588 (3.2)
	NTE	431 (2.3)
	NTW	381 (2.1)
	KC	349 (1.9)
	Unknown	64 (0.3)
Index event		
	Myocardial infarction	3637 (19.7)
	PCI	4096 (22.2)
	Stroke or TIA	10684 (58.0)
Year of diagnosis (%)		
	2003	349 (1.9)
	2004	1366 (7.4)
	2005	1453 (7.9)
	2006	1411 (7.7)
	2007	1455 (7.9)
	2008	1506 (8.2)
	2009	1573 (8.5)
	2010	1596 (8.7)
	2011	1666 (9.0)
	2012	1628 (8.8)
	2013	1589 (8.6)
	2014	1104 (6.0)
	2015	902 (4.9)
	2016	819 (4.4)
Length of index admission, days (mean (SD))		7.7 (14.7)
Laboratory tests		
	LDL-C, mmol/L (mean (SD))	2.8 (1.0)
	Total cholesterol, mmol/L (mean (SD))	4.7 (1.1)
	HDL-C, mmol/L (mean (SD))	1.2 (0.4)
	non–HDL-C, mmol/L (mean (SD))	3.5 (1.1)
	Triglycerides, mmol/L (median [IQR])	1.2 [0.9, 1.6]
	MDRD eGFR (mL/min/1.73 $m^{2}$) (mean (SD))	69.6 (26.0)
Diagnoses and procedures		
	Hypertension (%)	10388 (56.4)
	Diabetes mellitus (%)	5876 (31.9)
	Heart failure (%)	1585 (8.6)
	Smoking (%)	342 (1.9)
	Kidney disease (%)	981 (5.3)
	Myocardial infarction (%)	471 (2.6)
	PCI (%)	531 (2.9)
	CABG (%)	272 (1.5)
	Stroke or TIA (%)	1156 (6.3)
	Peripheral artery disease (%)	270 (1.5)
Medications		
	Lipid-modifying drug (%)	12295 (66.8)
	Statin (%)	12082 (65.6)
	Nonstatin lipid-modifying drug (%)	690 (3.7)
	Statin and nonstatin lipid-modifying drug (%)	481 (2.6)
	Fibrate (%)	599 (3.3)
	Ezetimibe (%)	72 (0.4)
	PCSK9 inhibitor (%)	1 (0.0)
	Bile acid sequestrant (%)	22 (0.1)
	Antiplatelet drug (%)	15535 (84.4)
	Antihypertensive (%)	15613 (84.8)
	Antidiabetic drug (%)	5866 (31.9)
Statin drug (%)		
	Atorvastatin	2103 (17.4)
	Fluvastatin	89 (0.7)
	Pravastatin	6 (0.0)
	Rosuvastatin	1281 (10.6)
	Simvastatin	8603 (71.2)
Statin intensity (%)		
	Low	1859 (15.4)
	Moderate	8463 (70.0)
	High	1760 (14.6)
TRS 2°P risk factors		
	Number (median [IQR])	2.0 [1.0, 3.0]
	0 risk factors	1201 (6.5)
	1 risk factor	4804 (26.1)
	2 risk factors	5576 (30.3)
	≥3 risk factors	6836 (37.1)

CABG, coronary artery bypass graft surgery; eGFR, estimated glomerular filtration rate; HDL-C, high-density lipoprotein cholesterol; HKW, Hong Kong West; HKE, Hong Kong East; IQR, interquartile range; KC, Kowloon Central; KE, Kowloon East; KW, Kowloon West; LDL-C, low-density lipoprotein cholesterol; MDRD, Modification of Diet in Renal Disease; non–HDL-C, non–high-density lipoprotein cholesterol; NTW, New Territories West; NTE, New Territories East; PCI, percutaneous coronary intervention; PCSK9, Proprotein Convertase Subtilisin/Kexin type 9; SD, standard deviation; TIA, transient ischaemic attack; TRS 2ºP, TIMI (Thrombolysis in Myocardial Infarction) Risk Score for Secondary Prevention.

**Fig. 2.**
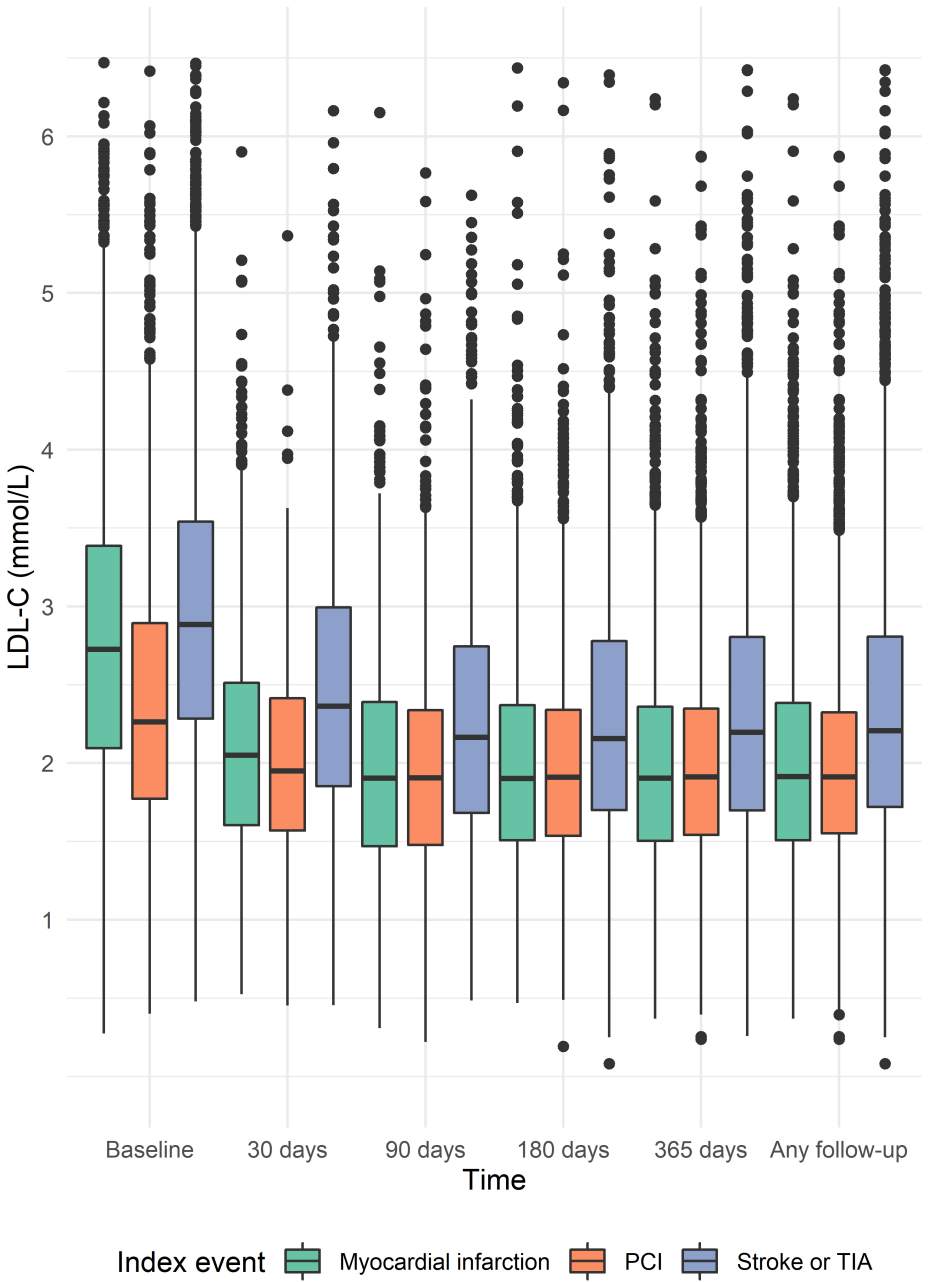
**Distribution of low-density lipoprotein cholesterol concentrations stratified by index event**. We assessed low-density lipoprotein concentrations at baseline (during the index hospitalization), and at 30, 90, 180, and 365 days after hospital discharge, or any time during one year of follow-up.

For each time window, the distribution of LDL-C stratified by index event is shown in Fig. 2. Most patients had an LDL-C result available at hospital discharge and during follow-up (**Supplementary Table 2**). The proportion of patients with a baseline LDL-C was 75.9% among patients with MI, 52.7% with PCI, and 83.0% with stroke or TIA. Overall, 1849 (10.0%) of patients had an LDL-C <1.8 mmol/L at baseline. By one year after discharge, a total of 4591 (24.9%) achieved an LDL-C <1.8 mmol/L. The proportion of patients achieving an LDL-C <1.8 mmol/L at any time during the one-year follow-up period was 35.4% among patients with MI, 37.6% with PCI, and 16.5% with stroke or TIA. A larger proportion of patients with PCI (89.5%) or MI (83.4%) had follow-up LDL-C results available any time during follow-up when compared to patients with an index stroke or TIA (56.8%).

The number of patients with a hospital discharge LDL-C concentration ≥1.8 mmol/L who subsequently achieved an LDL-C concentration of <1.8 mmol/L by 365 days after hospital discharge are presented in Table 2. Of the patients who had any baseline LDL-C result (n = 13783), 11934 (86.6%) had a baseline LDL-C concentration ≥1.8 mmol/L. Overall during follow-up, 18.6% of these patients achieved an LDL-C of <1.8 mmol/L. The proportion of patients who achieved this goal by the end of 365 days varied by index event. Fewer patients with a stroke or TIA and a baseline LDL-C ≥1.8 mmol/L achieved an LDL-C of <1.8 mmol/L one year after hospital discharge.

**Table 2.** **Number of patients with a baseline low-density lipoprotein cholesterol concentration ≥1.8 mmol/L, and the number and percentage of patients whose latest achieved low-density lipoprotein cholesterol concentration was <1.8 mmol/L any time after hospital discharge. Data are shown for the overall cohort and stratified by index event**.

	Number with LDL-C ≥1.8 mmol/L at baseline	Number (%) with follow-up LDL-C <1.8 mmol/L
Overall	11934	2214 (18.6)
Stroke or TIA	7981	1069 (13.4)
Myocardial infarction	2359	725 (30.7)
PCI	1594	420 (26.3)

LDL-C, low-density lipoprotein cholesterol; PCI, percutaneous coronary intervention; TIA, transient ischaemic attack.

Table 3 presents the baseline characteristics and percent reduction of LDL-C from baseline, stratified according to an LDL-C of 1.8 mmol/L by 365 days after hospital discharge, after excluding 5654 patients missing a follow-up LDL-C test. A larger proportion of patients who achieved an LDL-C of <1.8 mmol/L were prescribed statins at baseline as compared to patients who had an LDL-C ≥1.8 mmol/L (87.2% vs 67.6%). About 5% of patients in both groups used nonstatin therapies. Patients who achieved an LDL-C <1.8 mmol/L appeared to be at higher cardiovascular risk as 42.6% had ≥3 TRS $2^{\circ }$P risk factors.

**Table 3.** **Baseline characteristics and percent reductions in low-density lipoprotein cholesterol stratified by whether a patient achieved a low-density lipoprotein cholesterol level of <1.8 mmol/L by one year after hospital discharge**.

Characteristic		<1.8 mmol/L (n = 4591)	≥1.8 mmol/L (n = 8172)
Male (%)		3058 (66.6)	4990 (61.1)
Age, years (mean (SD))		69.7 (12.3)	68.2 (12.3)
Index event (%)			
	Myocardial infarction	1288 (28.1)	1744 (21.3)
	PCI	1542 (33.6)	2122 (26.0)
	Stroke or TIA	1761 (38.4)	4306 (52.7)
Percent reduction in LDL-C from baseline (mean (SD))		–35.5 (27.7)	–8.7 (35.4)
LDL-C, mmol/L (mean (SD))		2.5 (0.9)	3.1 (1.0)
Total cholesterol, mmol/L (mean (SD))		4.3 (1.1)	4.9 (1.2)
HDL-C, mmol/L (mean (SD))		1.1 (0.4)	1.2 (0.4)
non–HDL-C, mmol/L (mean (SD))		3.2 (1.0)	3.7 (1.1)
Triglycerides, mmol/L (median [IQR])		1.2 [0.9, 1.7]	1.3 [0.9, 1.7]
MDRD eGFR (mL/min/1.73 $m^{2}$) (mean (SD))		67.3 (25.9)	69.9 (25.4)
Hypertension (%)		2745 (59.8)	4577 (56.0)
Diabetes mellitus (%)		1916 (41.7)	2718 (33.3)
Heart failure (%)		468 (10.2)	697 (8.5)
Smoking (%)		125 (2.7)	159 (1.9)
Kidney disease (%)		317 (6.9)	451 (5.5)
Myocardial infarction (%)		148 (3.2)	220 (2.7)
PCI (%)		142 (3.1)	289 (3.5)
CABG (%)		103 (2.2)	144 (1.8)
Stroke or TIA (%)		242 (5.3)	510 (6.2)
Peripheral artery disease (%)		87 (1.9)	110 (1.3)
Lipid-modifying drug (%)		4032 (87.8)	5658 (69.2)
Statin (%)		4005 (87.2)	5525 (67.6)
Nonstatin lipid-modifying drug (%)		205 (4.5)	381 (4.7)
Statin and nonstatin lipid-modifying drug (%)		178 (3.9)	251 (3.1)
Fibrate (%)		177 (3.9)	323 (4.0)
Ezetimibe (%)		23 (0.5)	44 (0.5)
PCSK9 inhibitor (%)		0 (0.0)	1 (0.0)
Bile acid sequestrant (%)		6 (0.1)	16 (0.2)
Antiplatelet drug (%)		4261 (92.8)	6923 (84.7)
Antihypertensive (%)		4178 (91.0)	7078 (86.6)
Antidiabetic drug (%)		1942 (42.3)	2735 (33.5)
Statin drug (%)			
	Atorvastatin	728 (18.2)	1033 (18.7)
	Fluvastatin	12 (0.3)	45 (0.8)
	Pravastatin	1 (0.0)	5 (0.1)
	Rosuvastatin	628 (15.7)	525 (9.5)
	Simvastatin	2636 (65.8)	3917 (70.9)
Statin intensity (%)			
	Low	542 (13.5)	870 (15.7)
	Moderate	2694 (67.3)	3856 (69.8)
	High	769 (19.2)	799 (14.5)
TRS 2°P risk factors			
	Number (median [IQR])	2.0 [1.0, 3.0]	2.0 [1.0, 3.0]
	0 risk factors	162 (3.5)	471 (5.8)
	1 risk factor	1052 (22.9)	2332 (28.5)
	2 risk factors	1420 (30.9)	2519 (30.8)
	≥3 risk factors	1957 (42.6)	2850 (34.9)

ABG, coronary artery bypass graft surgery; eGFR, estimated glomerular filtration rate; HDL-C, high-density lipoprotein cholesterol; IQR, interquartile range; LDL-C, low-density lipoprotein cholesterol; MDRD, Modification of Diet in Renal Disease; non–HDL-C, non–high-density lipoprotein cholesterol; PCI, percutaneous coronary intervention; PCSK9, Proprotein Convertase Subtilisin/Kexin type 9; SD, standard deviation; TIA, transient ischaemic attack; TRS 2ºP, TIMI (Thrombolysis in Myocardial Infarction) Risk Score for Secondary Prevention.

Achieved LDL-C targets during follow-up, the distribution of LDL-C, and the percent reduction in LDL-C according to index year are shown in Table 4. Despite a similar percent reduction in LDL-C over the study period, the absolute mean achieved LDL-C concentrations, and the proportion of patients achieving either an LDL-C <1.8 mmol/L, ≥50% LDL-C reduction from baseline, or both, appears to increase over time.

**Table 4.** **Number of patients with a follow-up, and an in-hospital and follow-up low-density lipoprotein cholesterol level, and achieved low-density lipoprotein cholesterol one year after discharge, by year of index diagnosis. Percent reduction in low-density lipoprotein cholesterol could only be calculated for patients with an in-hospital and a follow-up low-density lipoprotein cholesterol level**.

Index year	Number with follow-up LDL-C	Number (%) with follow-up LDL-C <1.8 mmol/L	Number with in-hospital and follow-up LDL-C	Number (%) with ≥50% LDL-C reduction	Number (%) with ≥50% LDL-C reduction and LDL-C <1.8 mmol/L	Mean (SD) follow-up LDL-C, mmol/L	Mean LDL-C % reduction
2003	290	58 (20.0)	44	5 (11.4)	2 (4.5)	2.64 (0.92)	–17.7
2004	777	173 (22.3)	435	62 (14.3)	31 (7.1)	2.46 (0.87)	–14.4
2005	821	187 (22.8)	526	68 (12.9)	43 (8.2)	2.50 (0.90)	–14.9
2006	790	175 (22.2)	544	69 (12.7)	47 (8.6)	2.41 (0.82)	–13.9
2007	874	207 (23.7)	623	64 (10.3)	45 (7.2)	2.38 (0.80)	–14.1
2008	973	299 (30.7)	715	92 (12.9)	66 (9.2)	2.23 (0.77)	–17.7
2009	1100	369 (33.5)	752	126 (16.8)	86 (11.4)	2.15 (0.72)	–20.0
2010	1138	455 (40.0)	697	142 (20.4)	109 (15.6)	2.09 (0.77)	–21.3
2011	1258	490 (39.0)	809	149 (18.4)	116 (14.3)	2.06 (0.74)	–20.8
2012	1247	506 (40.6)	821	165 (20.1)	123 (15.0)	2.03 (0.71)	–20.8
2013	1197	545 (45.5)	781	167 (21.4)	141 (18.1)	1.97 (0.71)	–22.6
2014	891	438 (49.2)	507	102 (20.1)	89 (17.6)	1.93 (0.71)	–21.4
2015	752	336 (44.7)	450	73 (16.2)	66 (14.7)	1.96 (0.67)	–15.4
2016	655	353 (53.9)	425	63 (14.8)	56 (13.2)	1.86 (0.70)	–14.7

LDL-C, low-density lipoprotein cholesterol.

## 4. Discussion

Using a large cohort of Chinese individuals, we described LDL-C levels in-hospital and one year after discharge, and report important clinical characteristics such as the use of statin and nonstatin lipid-modifying drugs and the TRS 2ºP. During one year of follow-up, 75% of patients did not achieve an LDL-C <1.8 mmol/L. When examined by year of index event, average LDL-C levels declined and the proportion of patients achieving an LDL-C <1.8 mmol/L increased between 2003 to 2016. Despite the availability of multiple lipid-modifying treatments, we observed that about 1–2% of patients had follow-up LDL-C levels ≥4 mmol/L and 30% did not have a one-year follow-up LDL-C test result available in CDARS.

Our findings align with a previous study of patients with acute coronary syndrome who underwent PCI in Hong Kong between 2009 to 2015. Wang *et al*. [10] found that of these patients 11.3% received high-intensity statins, 26.8% did not receive a statin, and only 0.2% received ezetimibe. The proportion of patients with MI and PCI with a baseline and follow-up LDL-C <1.8 mmol/L was also similar to the findings of our study at about 12% and 35–40% respectively. Notably, despite that we followed-up patients until the end of 2017, 45% of patients did not receive a statin before hospital discharge, and there was limited use of ezetimibe and PCSK9 inhibitors.

We were particularly interested in the availability of in-hospital and follow-up LDL-C levels; missing lipid test patterns could identify gaps in monitoring patient response to lipid treatment. When examining the availability of LDL-C tests at baseline and during follow-up, a similar number of patients had an LDL-C level during their index hospitalization, but the proportion with follow-up levels increased from around 55% in 2004 to 80% in 2016. Further investigation is needed to understand the delay in obtaining follow-up LDL-C levels as nearly 30% of patients did not have a measured LDL-C within one year of the index event. It is possible that a group of patients with missing LDL-C values transfer their care to the private sector, leave Hong Kong, or obtain a follow-up LDL-C after 12 months, and would thus have a missing follow-up LDL-C result in our analysis.

Differences in achieved LDL-C levels exist when comparing Hong Kong to other countries. LDL-C levels during follow-up may have narrower distribution in our cohort compared with data from the United Kingdom. About 1.8% of patients in this study had LDL-C levels which remained >4 mmol/L during follow-up, but the proportion was generally less than those reported by Danese *et al*. [19], which ranged from 1.4% to 4.1%, in a similar cohort study that examined data from the Clinical Practice Research Datalink and the Hospital Episode Statistics. While difficult to make direct comparisons, a similar percentage of patients in the United Kingdom (23 to 42%) achieved an LDL-C <1.8 mmol/L at one-year after discharge [19], to those in our study (17% for patients with an index stroke or TIA to 38% for patients with an index PCI). Evidence from Italy, that assessed patients defined as very high risk according to ESC/EAS clinical guidelines between March 2016 to February 2017, demonstrates more frequent use of statins (94.1%), high-intensity statins (54.9%), and ezetimibe (14.4%) [20]. In this study, 58.1% of patients achieved a target LDL-C of <1.8 mmol/L [20]. A higher rate of LDL-C <1.8 mmol/L achievement in Italy corresponds to the greater prescribing prevalence of high-intensity statins, which was much lower in our study in Hong Kong.

This study has several limitations. Although calculated LDL-C remains the primary target for lipid modification in clinical practice guidelines, it becomes more inaccurate at low LDL-C and high triglyceride values [5]. The assessment of lipid-modifying drug effectiveness using the Friedewald formula could be confounded in some individuals because of the lack of fitness of calculated LDL-C in these contexts. The TRS 2ºP has only been validated in patients following acute coronary syndrome, and not with a diagnosis of stroke or TIA, thus the risk predictors of TRS 2ºP may not be completely applicable to all patients in our cohort. Initial lipid test data were identified only for one hospital. The Queen Mary Hospital is an important referral hospital in Hong Kong, which could cause selection bias of more severe or complex cases. CDARS only includes data from the Hospital Authority, and thus excludes private healthcare data. For example, after discharge patients could obtain follow-up prescriptions and laboratory testing at private hospitals or clinics. Finally, selection bias may also occur for patients who die after hospital discharge and prior to their first follow-up LDL-C result. However, only 913 patients died and had no recorded follow-up LDL-C level, which represents only 16% of the patients with no follow-up LDL-C results. The eligibility criteria were limited to patients who had a lipid test at a single hospital and we did not differentiate haemorrhagic and ischaemic stroke in our inclusion criteria, resulting in a more heterogeneous group of patients with stroke. To address these limitations, our research group is currently conducting an up-to-date assessment of LDL-C target achievement in all patients diagnosed with ASCVD (i.e., ischaemic heart disease, ischaemic stroke, TIA, and peripheral vascular disease) in the Hospital Authority from 2010 to 2020, regardless of whether a lipid test has been measured.

## 5. Conclusions

Between 2003 to 2016, there was vast under-treatment of ASCVD with lipid-modifying drugs in Hong Kong, with only 25% of patients in this study achieving a contemporary target LDL-C of <1.8 mmol/L. Local quality improvement initiatives and earlier uptake of both statin and nonstatin lipid-modifying drugs could help further reduce LDL-C and ultimately contribute to reducing the risk of recurrent cardiovascular events.

## Supplementary Material

Supplementary material associated with this article can be found, in the online version, at https://doi.org/10.31083/j.rcm2310327.
