# Supplementary material for: Low-Density Lipoprotein Cholesterol Treatment Target Achievement in Patients with Myocardial Infarction, Percutaneous Coronary Intervention, or Stroke in Hong Kong
Source: Rev Cardiovasc Med. 2022 Sep 26;23(10):327. doi: 10.31083/j.rcm2310327 (PMC11267373; doi:10.31083/j.rcm2310327)
Supplement: Supplementary file 1 [file 2153-8174-23-10-327-s1.docx]

**Supplementary online material**

**Supplementary Table 1.** Details of variables used in the study. Note that all time windows either refer to calendar time or days before or after time zero (index date), and square brackets indicate inclusive.

| **Variable category** | **Variable name** | **Definition** | **Assessment Window** | **Comments** |
| --- | --- | --- | --- | --- |
| ***Diagnoses and procedures*** | | ***ICD-9-CM Code/ICPC Code*** |  |  |
| **Index event** | Myocardial infarction | 410 | 1 January 2003 - 31 December 2016 | Earliest discharge date for inpatient diagnosis or procedures in D1/P1 to D3/P3 position |
|  | Stroke or transient ischaemic attack | 430, 431, 432, 433.01, 433.11, 433.21, 433.31, 433.81, 433.91, 434, 435, 436, 437.0, 437.1 |  |  |
|  | Percutaneous coronary intervention | 36.01-36.02, 36.05-36.07 |  |  |
| **Baseline diagnoses and procedures** | Hypertension | 401-405  K86, K87 | [-365,0] | Any position in either all diagnosis, inpatient diagnosis, outpatient diagnosis |
|  | Diabetes mellitus | 250  T89, T90, T901, W85 | [-365,0] | Any position in either all diagnosis, inpatient diagnosis, outpatient diagnosis |
|  | Heart failure | 428  K77 | [-365,0] | Any position in either all diagnosis, inpatient diagnosis, outpatient diagnosis |
|  | History of coronary artery bypass graft surgery | 36.10-36.17, 36.19, 36.2 | [Start of procedure data (1993),0] | Procedure codes.  Any position in either inpatient diagnosis or all procedures |
|  | Prior stroke or TIA | 430, 431, 432, 433.01, 433.11, 433.21, 433.31, 433.81, 433.91, 434, 435, 436, 437.0, 437.1 | [Start of diagnosis data (1993),index admission date) | Do not include the index admission diagnoses.  Any position in either all diagnosis or inpatient diagnosis |
|  | Prior MI | 410 | [Start of diagnosis data (1993), index admission date- | Do not include the index admission diagnoses.  Any position in either all diagnosis or inpatient diagnosis |
|  | Prior PCI | 36.01-36.02, 36.05-36.07 | [Start of procedure data (1993),index admission date)] | Do not include the index admission diagnoses.  Any position in either inpatient diagnosis. Procedure date should occur prior to the index admission |
|  | Smoker | 305.1, V15.82, V15.83, 649.0  P17 | [-365,0] | Any position in either all diagnosis, inpatient diagnosis, outpatient diagnosis |
|  | Kidney disease | 403.01, 403.11, 403.91, 404.02, 404.03, 404.12, 404.13, 404.92, 404.93, 582, 583.0-583.7, 585, 586, 588.0, V42.0, V45.1, V56  U99 | [-365,0] | Any position in either all diagnosis, inpatient diagnosis, outpatient diagnosis |
|  | Peripheral artery disease | 440, 443.9  K92 | [-365,0] | Any position in either all diagnosis, inpatient diagnosis, outpatient diagnosis |
| ***Prescriptions*** |  | ***BNF Section/Drug Name*** |  |  |
| **Baseline prescriptions** | Lipid-modifying drugs | 2.12 | [-365,0] |  |
|  | Antiplatelet drugs | 2.9 | [-365,0] |  |
|  | Antihypertensive drugs | 2.2.3, 2.2.4, 2.4, 2.5.2, 2.5.4, 2.5.5.1, 2.5.5.2, 2.5.5.3, 2.6.2 | [-365,0] |  |
|  | Antidiabetic drugs | 6.1.1.1, 6.1.1.2, 6.1.2.1, 6.1.2.2, 6.1.2.3 | [-365,0] |  |
|  | Statins | atorvastatin (also caduet)  rosuvastatin  simvastatin (also vytorin)  pravastatin  lovastatin  fluvastatin | [-365,0] |  |
|  | Statins, high-intensity | atorvastatin >20 mg/day  rosuvastatin >10 mg/day  simvastatin >40 mg/day | [-365,0] |  |
|  | Statins, moderate-intensity | atorvastatin ≤20 mg/day  rosuvastatin ≤10 mg/day simvastatin >10 to ≤40 mg/day  pravastatin >20 mg/day  lovastatin >20 mg/day  fluvastatin >40 mg/day | [-365,0] |  |
|  | Statins, low-intensity | simvastatin ≤10 mg/day  pravastatin ≤20 mg/day  lovastatin ≤20 mg/day  fluvastatin ≤40 mg/day | [-365,0] |  |
|  | Ezetimibe | ezetimibe (also Vytorin) | [-365,0] |  |
|  | Fibrate | bezafibrate  fenofibrate  gemfibrozil | [-365,0] |  |
|  | Bile acid sequestrants | cholestyramine | [-365,0] |  |
|  | PCSK9 inhibitor | alirocumab  evolocumab | [-365,0] |  |
|  | Statin and nonstatin | Prescription for a statin and a nonstatin (i.e. ezetimibe, fibrate, bile acid sequestrant, PCSK9 inhibitor) | [-365,0] |  |
| **TIMI Risk Score** | TRS 2°P hypertension | Either a diagnosis code of hypertension or an antihypertensive drug prescription | [-365,0] |  |
|  | TRS 2°P diabetes mellitus (DM) | Either a diagnosis code of diabetes or an antidiabetic drug prescription | [-365,0] |  |
|  | TRS 2°P age | Age on index date ≥ 75 | [0,0] |  |
|  | TRS 2°P eGFR <60 ml/min | Present if measured creatinine clearance is < 60, or estimated eGFR (MDRD) is < 60 or presence of kidney disease covariate (diagnosis codes) | [-365,0] | Use lab tests for creatinine clearance or serum creatinine first, then diagnosis of kidney disease. |
|  | Baseline TIMI risk score for secondary prevention | Sum all risk factors (use specific TRS 2°P covariates for classification of diabetes mellitus, hypertension, age, and eGFR) |  |  |
|  | TRS 2°P categories | [0,1), [1,2), [2,3), [3,8] |  |  |
| **Demographics** | Hospital authority cluster of residence |  | Closest to index date | Selected from outpatient attendance nearest the index date. If multiple attendances, select the one on or prior index date. If missing from outpatient, select from A&E attendance on or nearest index date. |
|  | Nationality | Classified into Chinese, other, or missing (missing or recorded as unknown) |  |  |
|  | Age on index date | Difference between date of birth and index date / 365.25 | Index date |  |
|  | Sex | M/F | Index date |  |
| **Laboratory tests** | Baseline total cholesterol | mmol/L | [index admission date,0] | Latest result in window. If multiple results, arithmetic mean of results nearest index date. |
|  | Baseline HDL-C | mmol/L |  |  |
|  | Baseline non-HDL-C | mmol/L; if missing calculated as total cholesterol − HDL-C |  |  |
|  | Baseline triglycerides | mmol/L |  |  |
|  | Baseline serum creatinine | umol/L | [-365,0] |  |
|  | Baseline creatinine clearance | mL/min | [-365 0] |  |
|  | One LDL-C result within inclusion window |  | [index admission date,365] | Inclusion criteria |
| **Exposure** | Baseline LDL-C | mmol/L | [index admission date,0] | Latest result in window. If multiple results, arithmetic mean of results nearest index date. |
| **Outcomes** | LDL-C concentration within each follow-up window | mmol/L | [1,30] (30 days)  [31,90] (90 days)  [91,180] (180 days)  [181,365] (365 days)  [1,365] (any time) | Latest result in window. If multiple results, arithmetic mean of results farthest away from index date. |
|  | Percent LDL-C change at one year after discharge | (LDL-C at one year – baseline LDL-C) / Baseline LDL-C * 100% |  | Calculated for each individual. If no baseline or follow-up LDL-C results, code as missing |

Abbreviations: eGFR, estimated glomerular filtration rate; HDL-C, high-density lipoprotein cholesterol; ICD-9-CM, International Classification of Disease, 9^th^ Revision Clinical Modification; ICPC, International Classification of Primary Care; LDL-C, low-density lipoprotein cholesterol; MDRD, Modification of Diet in Renal Disease; MI, myocardial infarction; non-HDL-C, non-high-density lipoprotein cholesterol; PCI, percutaneous coronary intervention; PCSK9, proprotein convertase subtilisin/kexin type 9; TIA, transient ischaemic attack; TRS 2ºP, TIMI (Thrombolysis in Myocardial Infarction) Risk Score for Secondary Prevention.

**Supplementary Table 2.** Distribution and availability of low-density lipoprotein cholesterol concentrations for the overall cohort and stratified by index event. We assessed low-density lipoprotein concentrations at baseline (during the index hospitalization), and at 30, 90, 180, and 365 days after hospital discharge, or at any time during one year of follow-up.

| **Time** | **Overall (n=18 417)** | **Myocardial infarction (n=3 637)** | **PCI (n=4 096)** | **Stroke or TIA (n=10 684)** |
| --- | --- | --- | --- | --- |
| Mean (SD) LDL-C, mmol/L | | | | |
| Index hospitalization | 2.8 (1.0) | 2.8 (1.0) | 2.4 (0.9) | 3.0 (1.0) |
| 30 days | 2.3 (0.9) | 2.1 (0.8) | 2.0 (0.8) | 2.5 (0.9) |
| 90 days | 2.1 (0.8) | 2.0 (0.8) | 2.0 (0.8) | 2.3 (0.8) |
| 180 days | 2.1 (0.8) | 2.0 (0.7) | 2.0 (0.8) | 2.3 (0.8) |
| 365 days | 2.1 (0.8) | 2.0 (0.7) | 2.0 (0.7) | 2.3 (0.8) |
| Any time during follow-up | 2.2 (0.8) | 2.0 (0.7) | 2.0 (0.7) | 2.3 (0.8) |
| Category of LDL-C | | | | |
| Index hospitalization | | | | |
| <1.8 mmol/L | 1 849 (10.0) | 402 (11.1) | 563 (13.7) | 884 (8.3) |
| 1.8-2.6 mmol/L | 4 688 (25.5) | 945 (26.0) | 909 (22.2) | 2 834 (26.5) |
| 2.7-3.9 mmol/L | 5 647 (30.7) | 1 097 (30.2) | 575 (14.0) | 3 975 (37.2) |
| ≥4 mmol/L | 1 599 (8.7) | 317 (8.7) | 110 (2.7) | 1 172 (11.0) |
| Missing | 4 634 (25.2) | 876 (24.1) | 1 939 (47.3) | 1 819 (17.0) |
| 30 days | | | | |
| <1.8 mmol/L | 676 (3.7) | 266 (7.3) | 165 (4.0) | 245 (2.3) |
| 1.8-2.6 mmol/L | 987 (5.4) | 359 (9.9) | 185 (4.5) | 443 (4.1) |
| 2.7-3.9 mmol/L | 481 (2.6) | 119 (3.3) | 53 (1.3) | 309 (2.9) |
| ≥4 mmol/L | 102 (0.6) | 22 (0.6) | 4 (0.1) | 76 (0.7) |
| Missing | 16 171 (87.8) | 2 871 (78.9) | 3 689 (90.1) | 9 611 (90.0) |
| 90 days | | | | |
| <1.8 mmol/L | 1 243 (6.7) | 436 (12.0) | 370 (9.0) | 437 (4.1) |
| 1.8-2.6 mmol/L | 1 355 (7.4) | 407 (11.2) | 372 (9.1) | 576 (5.4) |
| 2.7-3.9 mmol/L | 567 (3.1) | 150 (4.1) | 99 (2.4) | 318 (3.0) |
| ≥4 mmol/L | 81 (0.4) | 18 (0.5) | 17 (0.4) | 46 (0.4) |
| Missing | 15 171 (82.4) | 2 626 (72.2) | 3 238 (79.1) | 9 307 (87.1) |
| 180 days | | | | |
| <1.8 mmol/L | 2 051 (11.1) | 679 (18.7) | 756 (18.5) | 616 (5.8) |
| 1.8-2.6 mmol/L | 2 349 (12.8) | 654 (18.0) | 789 (19.3) | 906 (8.5) |
| 2.7-3.9 mmol/L | 899 (4.9) | 199 (5.5) | 221 (5.4) | 479 (4.5) |
| ≥4 mmol/L | 134 (0.7) | 26 (0.7) | 23 (0.6) | 85 (0.8) |
| Missing | 12 984 (70.5) | 2 079 (57.2) | 2 307 (56.3) | 8 598 (80.5) |
| 365 days | | | | |
| <1.8 mmol/L | 3 261 (17.7) | 938 (25.8) | 1 206 (29.4) | 1 117 (10.5) |
| 1.8-2.6 mmol/L | 3 723 (20.2) | 937 (25.8) | 1 236 (30.2) | 1 550 (14.5) |
| 2.7-3.9 mmol/L | 1 525 (8.3) | 250 (6.9) | 350 (8.5) | 925 (8.7) |
| ≥4 mmol/L | 212 (1.2) | 37 (1.0) | 38 (0.9) | 137 (1.3) |
| Missing | 9 696 (52.6) | 1 475 (40.6) | 1 266 (30.9) | 6 955 (65.1) |
| Any time during follow-up | | | | |
| <1.8 mmol/L | 4 591 (24.9) | 1 288 (35.4) | 1 542 (37.6) | 1 761 (16.5) |
| 1.8-2.6 mmol/L | 5 520 (30.0) | 1 310 (36.0) | 1 634 (39.9) | 2 576 (24.1) |
| 2.7-3.9 mmol/L | 2 328 (12.6) | 382 (10.5) | 440 (10.7) | 1 506 (14.1) |
| ≥4 mmol/L | 324 (1.8) | 52 (1.4) | 48 (1.2) | 224 (2.1) |
| Missing | 5 654 (30.7) | 605 (16.6) | 432 (10.5) | 4 617 (43.2) |
| Achieved LDL-C of < 1.8 mmol/L | | | | |
| Index hospitalization | | | | |
| <1.8 mmol/L | 1 849 (10.0) | 402 (11.1) | 563 (13.7) | 884 (8.3) |
| ≥1.8 mmol/L | 11 934 (64.8) | 2 359 (64.9) | 1 594 (38.9) | 7 981 (74.7) |
| Missing | 4 634 (25.2) | 876 (24.1) | 1 939 (47.3) | 1 819 (17.0) |
| 30 days | | | | |
| <1.8 mmol/L | 676 (3.7) | 266 (7.3) | 165 (4.0) | 245 (2.3) |
| ≥1.8 mmol/L | 1 570 (8.5) | 500 (13.7) | 242 (5.9) | 828 (7.7) |
| Missing | 16 171 (87.8) | 2 871 (78.9) | 3 689 (90.1) | 9 611 (90.0) |
| 90 days | | | | |
| <1.8 mmol/L | 1 243 (6.7) | 436 (12.0) | 370 (9.0) | 437 (4.1) |
| ≥1.8 mmol/L | 2 003 (10.9) | 575 (15.8) | 488 (11.9) | 940 (8.8) |
| Missing | 15 171 (82.4) | 2 626 (72.2) | 3 238 (79.1) | 9 307 (87.1) |
| 180 days | | | | |
| <1.8 mmol/L | 2 051 (11.1) | 679 (18.7) | 756 (18.5) | 616 (5.8) |
| ≥1.8 mmol/L | 3 382 (18.4) | 879 (24.2) | 1 033 (25.2) | 1 470 (13.8) |
| Missing | 12 984 (70.5) | 2 079 (57.2) | 2 307 (56.3) | 8 598 (80.5) |
| 365 days | | | | |
| <1.8 mmol/L | 3 261 (17.7) | 938 (25.8) | 1 206 (29.4) | 1 117 (10.5) |
| ≥1.8 mmol/L | 5 460 (29.6) | 1 224 (33.7) | 1 624 (39.6) | 2 612 (24.4) |
| Missing | 9 696 (52.6) | 1 475 (40.6) | 1 266 (30.9) | 6 955 (65.1) |
| Any time during follow-up | | | | |
| <1.8 mmol/L | 4 591 (24.9) | 1 288 (35.4) | 1 542 (37.6) | 1 761 (16.5) |
| ≥1.8 mmol/L | 8 172 (44.4) | 1 744 (48.0) | 2 122 (51.8) | 4 306 (40.3) |
| Missing | 5 654 (30.7) | 605 (16.6) | 432 (10.5) | 4 617 (43.2) |
| LDL-C result available | | | | |
| Index hospitalization | 13 783 (74.8) | 2 761 (75.9) | 2 157 (52.7) | 8 865 (83.0) |
| 30 days | 2 246 (12.2) | 766 (21.1) | 407 (9.9) | 1 073 (10.0) |
| 90 days | 3 246 (17.6) | 1 011 (27.8) | 858 (20.9) | 1 377 (12.9) |
| 180 days | 5 433 (29.5) | 1 558 (42.8) | 1 789 (43.7) | 2 086 (19.5) |
| 365 days | 8 721 (47.4) | 2 162 (59.4) | 2 830 (69.1) | 3 729 (34.9) |
| Any time during follow-up | 12 763 (69.3) | 3 032 (83.4) | 3 664 (89.5) | 6 067 (56.8) |
| Abbreviations: LDL-C, low-density lipoprotein cholesterol; PCI, percutaneous coronary intervention; SD, standard deviation; TIA, transient ischaemic attack. All values are number (%) of patients unless otherwise indicated. | | | | |
